# FDA approval of copper histidinate (Zycubo) for Menkes disease: a therapeutic breakthrough in a rare pediatric neurodegenerative disorder

**DOI:** 10.1097/MS9.0000000000005071

**Published:** 2026-04-27

**Authors:** Muhammad Hammad Ghani, Muhammad Ahsan, Ahmed Asad Raza, Abedin Samadi

**Affiliations:** a Department of Medicine, Karachi Medical and Dental College, Karachi, Pakistan; b Department of Medicine, Jinnah Sindh Medical University, Karachi, Pakistan; c Department of Medicine, Kabul University of Medical Sciences, Abu Ali Ibn Sina, Kabul, Afghanistan

**Keywords:** ATP7A mutation, copper histidinate, Menkes disease, pediatric neurodegeneration, Zycubo therapy

## Abstract

Menkes disease (MD) is a rare X-linked recessive disorder caused by mutations in the ATP7A gene, leading to impaired copper transport and deficiency of essential copper-dependent enzymes. The disease is characterized by progressive neurodegeneration, refractory seizures, hypotonia, and connective tissue abnormalities, typically presenting within the first months of life. Without effective treatment, the prognosis remains poor, with most affected children dying before 3 years of age. Traditional therapeutic strategies have included oral copper salts; however, these approaches have shown limited clinical efficacy due to inadequate correction of systemic and cerebral copper deficiency. Parenteral copper histidinate therapy represented a significant advancement by improving copper bioavailability and enhancing systemic absorption. Early initiation of therapy, particularly within the first month of life, has been associated with improved survival and better neurodevelopmental outcomes in some patients. Recently, the U.S. Food and Drug Administration approved Zycubo (copper histidinate) as the first disease-specific treatment for children with MD, marking an important milestone in the management of this rare condition. Despite its clinical benefits, treatment may be associated with adverse effects including infections, respiratory complications, anemia, and injection-site reactions, necessitating careful monitoring during therapy. Continued research is required to evaluate long-term safety, optimize therapeutic strategies, and further improve outcomes for affected children.

*Dear Editor,*

Menkes disease (MD), also referred to as kinky hair disease, is an X-linked recessive disorder that leads to neural degeneration due to genetic alterations in the ATP7A gene. This gene encodes an enzyme that plays a crucial role in copper efflux from the cell and activates copper-dependent enzymes. Patients with classical MD present with neurodegeneration, which manifests as resistant seizures during the first months of life. Patients also experience neurodevelopmental regression. Children with MD present at the age of 3–6 months with symptoms such as low copper and ceruloplasmin levels, hypotonia, and the diagnosis is confirmed by genetic testing. Connective tissue dysfunction is another key feature of MD, which includes skeletal, vascular, skin, urogenital, and gastrointestinal anomalies^[^1^]^. The neurological and systemic symptoms develop due to the deficiency of essential copper enzymes, such as peptidylglycine α-amidating monooxygenase and dopamine-β-hydroxylase, which play a key role in the neurochemical regulation of the body^[^2^]^. It affects approximately one in every 100 000–250 000 live births worldwide^[^3^]^. Patients with MD have an average life expectancy of less than 3 years despite early treatment and care^[^1^]^.

The previously used treatment of MD involved oral copper salts, such as sulfate, acetate, and chloride, which were administered; however, these approaches failed to significantly correct serum copper and ceruloplasmin levels and showed limited clinical benefit. However, parenteral (injected) copper is used to induce the synthesis of apo-ceruloplasmin, which can increase serum copper and ceruloplasmin levels. Despite this, copper concentrations in the brain often remain unchanged, and clinical improvement is not always observed. The development of copper histidinate complexes represented a critical advancement, enhancing copper bioavailability and improving systemic absorption, and sometimes alleviating the symptoms of the disease^[^4^]^.

Recent studies indicate that treatment with copper histidinate in patients with MD can improve the transport of copper to the developing brain and lead to better clinical outcomes^[^2^]^. Children who received therapy within the first 4 weeks after birth showed a 78% decrease in mortality risk compared with those who remained untreated. Approximately half of the patients who received early therapy lived longer than 6 years, and some survived beyond 12 years, whereas none of the individuals in the untreated control group lived longer than 6 years^[^3^]^. Even with early diagnosis and treatment with CuHis initiated within the first 28 days of life (corrected for prematurity), the mortality rate under the age of five remains around 45%. In addition, although some treated patients may achieve normal or nearly normal neurodevelopmental outcomes, delays of variable scope continue to be reported in a proportion of individuals receiving CuHis treatment for MD^[^2^]^. The recent approval of Zycubo (copper histidinate) by the U.S. Food and Drug Administration marks the first disease-specific therapy for children with MD^[^3^]^. Furthermore, recent pharmaceutical advancements have focused on improving formulation stability and injectable quality standards to optimize therapeutic outcomes^[^5^]^.

Beyond its clinical benefits, several adverse effects have been reported with Zycubo, including infections, respiratory complications, anemia, vomiting, seizures, pyrexia, and reactions at the injection site. Given the risk of copper accumulation, vigilant monitoring is essential during therapy^[^3^]^.

In light of the current evidence, clinicians should practice an advanced approach for the early diagnosis of the disease, along with the timely administration of copper histidinate, to improve survival of the affected children, while taking into consideration the negative effects of copper histidinate. However, the long-term potency, safety, and durability of copper histidinate remain areas of continued investigation and should go toward a better option that has minimal side effects and higher efficiency.

To ensure methodological transparency and ethical compliance in manuscript preparation, we adhered to the recently developed TITAN (Transparency in the Reporting of Artificial Intelligence) 2025 guidelines, which outline standards for responsible AI use in biomedical writing and publishing^[^6^]^.

## Data Availability

No datasets were generated or analyzed during the current study.

## Research registration unique identifying number (UIN)

Not available.
